# Comparative Double Auction Approach for Peer-to-Peer Energy Trading on Multiple microgrids

**DOI:** 10.1007/s40866-023-00178-x

**Published:** 2023-11-21

**Authors:** Sweta Malik, Subhasis Thakur, Maeve Duffy, John G. Breslin

**Affiliations:** 1 Insight Centre for Data Analytics, University of Galway, Galway, Ireland; 2 Electrical and Electronic Engineering, School of Engineering, University of Galway, Galway, Ireland

**Keywords:** Peer-to-Peer energy trading, Prosumer, MGs, Double auction, Electricity market, Bidding strategies

## Abstract

Peer-to-peer (P2P) energy trading is one of the most effective methods to increase the usage of Renewable Energy (RE) resources in the distribution network and reduce losses by eliminating long transmission and distribution lines. This research aims to enhance the efficiency of P2P energy trading by examining the suitability of four distinct double auction mechanisms: Average, McAfee, Trade Reduction and Vickrey-Clarke-Groves (VCG). We conducted a systematic evaluation of these mechanisms across various microgrid (MG) types. The study algorithm integrates user preferences, bidding strategies and time-of-use tariffs, allowing participants to indicate their willingness to pay for different energy qualities and specific time periods. Notably, both the Average and VCG mechanisms emerged as the most effective across a majority of MG setups. Specifically, the average mechanism was found to be optimal for a consumer-centric MG, while the VCG mechanism was predominantly advantageous during non-peak hours trading. However, it was observed that P2P energy trading from MG to MG was inefficient due to the lesser number of peers. In conclusion, this work offers a comprehensive solution that adeptly identifies and recommends the most fitting auction mechanisms for diverse MG configurations and usage timings, paving the way for more efficient P2P energy trading.

## Introduction

With the ongoing paradigm shift from fossil fuels to Renewable Energy (RE), the electricity market is undergoing a transformation: evolving from a unidirectional system to a bidirectional competitive market. The technological advancements in Distributed Energy Resources (DER) and their increasing deployment in distribution networks are spurring consumers to transition from being consumers to prosumers [1]. Prosumers, individuals or entities connected to the main grid possess the capability to generate RE through systems like rooftop solar installations or other DERs. They can also store surplus energy using battery storage or in electric vehicles and have the potential to feed excess energy back to the grid or to other connected users. A substantial presence of prosumers in the network, armed with trading capabilities, combined with efficient scheduling, energy storage and efficient management systems, might present an effective way to harmonize energy demand and supply [2].

However, the prevailing feed-in tariffs that Distribution System Operators (DSO) offer to prosumers as compensation for selling excess energy after meeting their own demand are often not sufficiently incentivizing [3]. Net metering and power purchase agreements are common methods deployed to establish fixed-price contracts between prosumers and DSOs. As of the beginning of 2020, [4] reported that 70 countries had implemented national net metering laws. Meanwhile, others, such as the United States and Canada, operate under state-level net metering statutes. Nonetheless, these methods inadvertently pose challenges, acting as barriers for prosumers wishing to establish a centralized trading environment with consumers.

Addressing the mentioned challenges, Peer-to-Peer (P2P) energy trading emerges as a promising solution to motivate prosumers towards achieving higher revenue and enable consumers to cut down their electricity bills. P2P energy trading, as discussed in [5], stands out as an effective mechanism to increase the utilization of RE. Often displayed as the model of the shared economy, the P2P paradigm is primarily deployed within localized grid systems. By setting the energy selling price below the retail rates of the grid, prosumers are encouraged to maintain a balanced energy distribution. [6]. This strategy not only encourages them to sell their surplus energy amongst peers but also cultivates a win-win approach for both consumers and prosumers [6]. Additionally, P2P energy trading isn’t just beneficial for individual prosumers and consumers. Other market players in the electricity market stand to gain immensely. Benefits These include a decrease in peak demand, reduction in maintenance and operational expenses and improved reliability of the electrical system peak demand [7]. Further the P2P model contributes to the energy network’s resilience by reducing its dependency on centralized systems.

In recent years, the significance of energy trading in a bidirectional competitive energy market has garnered considerable attention. P2P trading mechanisms are principally categorized into three groups based on their foundations in game theory: those relying on cooperative game theory [8], non-cooperative game theory [9] and auction-based mechanisms [10]. The literature on P2P energy trading proposes several auction mechanisms for P2P energy trading, encompassing the uniform price auction, discriminatory auction, Double Auction (DA) and combinatorial auction. Each mechanism possesses distinct characteristics influencing its efficiency, fairness and overall performance. Our research emphasizes the use of DA in P2P energy trading. DA offers multiple advantages over other auction mechanisms within this context. Specifically, it enhances market efficiency by inviting both consumers and prosumers to engage in the auction, thereby intensifying competition and mitigating market dominance. Additionally, the DA can curtail transaction expenses by sidestepping intermediaries like DSOs and promoting direct interactions between consumers and prosumers. Due to its impressive scalability and efficiency, the DA is increasingly recognized as an optimal mechanism for energy trading.

While the DA presents numerous advantages, it is not without its challenges in the context of P2P energy trading. One notable concern is the inherent complexity of the DA, which can escalate computational costs and potentially reduce scalability. Such limitations may hinder its integration into real distribution systems. Another vulnerability of the DA is its susceptibility to strategic bidding. In such scenarios, bidders might exploit the system, forwarding misleading bids or offers to secure undeserved advantages. Moreover, the interconnection between auction mechanisms and bidding strategies remains a vibrant research area. A comprehensive understanding of their collective influence on P2P energy trading is yet to be achieved. Notably, the efficiency of various auction mechanisms can differ based on specific Microgrid (MG) attributes like size, topology and energy demand-supply dynamics. It becomes imperative, then, to assess auction mechanisms across diverse MG types to pinpoint those most capable for a given MG situation. This will require the development of new models and algorithms that can capture the unique features of different MGs and simulate their behaviour under various auction mechanisms.

### Related Work

The Continuous Double Auction (CDA) mechanism has been the subject of extensive study and application across diverse markets due to its real-time trading capabilities and inherent efficiency. A recent research by [11] investigated the potential of the CDA mechanism combined with Stackelberg games for intra-MG trading. This approach was introduced to address the computational and communicational limitations inherent to existing methodologies. Notably, results indicate that this method can effectively identify the substitution model swiftly and accurately represent market behaviours without increasing computational time as the network grows. In another influential study, the authors of [12] implemented a two-stage CDA for P2P trading to model prosumer behaviour and employ a deep neural network to guide dynamic pricing based on network constraints. Results highlight improved responsiveness and economic advantages, with a notable 5.12% decrease in power purchase costs. Furthermore, research by [13] utilized the CDA mechanism and model predictive control theory for optimizing flexible resource bidding and introduced an Automatic Learning (AL) pricing strategy, ensuring interactive learning among prosumers and price privacy. Despite the extensive research on CDA, few have explored that it may not provide the same level of control over price dynamics and resource allocation as compared to other double auctions. However, Discrete Double Auction (DDA) is often used in situations that require batch processing or where the precise allocation of limited resources is crucial, such as energy markets or commodity exchanges. A study on DDA by [14], adapting the McAfee’s mechanism, addressed the challenges posed by the rise in DER in P2P transactions. Using ensemble learning for energy forecasting and power flow optimization for optimal scheduling, this framework employs a trading mechanism for MGs. However, unlike other studies employing CDA, authors in [15] discussed into LEM using multi-agent-based simulations and DDA.

By employing a modified double auction mechanism on a virtual coin called Pcoin in [16], the ETradeChain platform is introduced for decentralized and equitable energy trading within LEM. The system employs blockchain technology to address security challenges in energy trading. Another work leveraging blockchain technology to innovate green power trading is presented in [17]. By introducing an improved double auction model, the study addresses the unique trading needs of decentralized, small-scale MGs, offering a more efficient and transparent peer-to-peer trading platform. A sophisticated three-layer architecture for P2P electricity trading is introduced in [18], recognizing the inherent transparency of blockchain and its potential risks to data privacy in P2P trades. Notably, results illustrate that the DA trading mechanism not only maintains data privacy but also securely manages trading operations. Another approach was proposed in [19], where a forward intra-day market using an auction mechanism was implemented on a blockchain network (Proof of Authority) that used discriminative prices for market clearing to calculate trading prices, considering multiple MGs.

To address the limitations of a centralized DA network, [20] introduced a system in which any peer can act as an auctioneer and receive multiple bids and sell prices. Should a peer be unable to fulfil auctioneer responsibilities, the algorithm transitions to another peer within the network. In a different approach, authors in [21] introduced a two-stage bidding strategy for P2P energy trading in residential MGs. Their strategy ensures fair competition, offers economic benefits for participants and promotes MG self-sufficiency. Additionally, they provide tools that empower residents to make informed decisions by introducing a trading price predictor and a risk analysis tool. The effectiveness of this proposed method is further illuminated through case studies. In the context of dynamic autonomous Electric Vehicle (EV) energy trading, the application of DA mechanisms was examined in [22]. Here, the authors devised an approach where auctioneers select EV winners from a cohort of players offering prices below the average. Building on this, a study by [23] evaluated optimal integration strategies for grid-connected EVs. It accentuated the potential of Vehicle-to-the-grid technology, which capitalizes on bidirectional power flow from EV batteries for either charging or supporting the utility grid. Exploring further, [24] introduced a P2P energy trading framework buttressed by bilateral agreements, a VCG mechanism and provisions for trading with the DSO. Finally, a study in [25] proposed a fully decentralized negotiation mechanism for P2P collective energy and reserve markets, grounded in the consensus alternating direction method of multipliers (ADMM) theory.

In a recent study by [26], the authors focused on a P2P market utilizing double auctions using four specific auction mechanisms: k-double, Vickrey-Clarke-Groves (VCG), McAfee and maximum volume matching (MVM). These four specific auction mechanisms were investigated and an automated bidding strategy using multi-agent, multi-armed bandit learning was proposed. While this approach proved efficient in simulating market designs, the research did not consider real-world physical network constraints, underscoring a limitation in its applicability and emphasizing the need for further refinement. Authors in [27] showcase how different approaches, specifically system marginal price, VCG and Pay-as-bid, can result in revenue imbalances in the market. To counteract this, compensation mechanisms that encourage specific peer behaviours are introduced. The findings indicate that these mechanisms can be harnessed to promote desired behaviours like investing in grid support or flexible energy usage. In [28], a comparative analysis of four auction mechanisms (Walrasian, VCG, MUDA Lottery and MUDA VCG) was conducted to illustrate their implementation with real-time data, showing the total gain from trade by all four mechanisms. Another comparative study of discriminatory and uniform k-Double Auction mechanisms was presented in [29], where the authors analyzed the amount of energy traded based on the percentage of penetration of solar PV using different bidding strategies, including random, preference and game theoretic model. In [30], the VCG double auction was compared with the linear-program-based perturbation mechanism, where each of them can eliminate the other on the basis of truthfulness. The study showed that while the VCG mechanism ensures truthful bidding, eliminates market power and maximizes Social Welfare (SW), it may cause a budget deficit, whereas the linear mechanism satisfies the budget balance property but at the cost of SW. A proposed CDA mechanism was compared with existing auction mechanisms such as the VCG mechanism, Trade Reduction Mechanism and clustering VCG auction in [31] and the authors found that only the CDA mechanism achieved all desired properties. However, it is essential to mention that previous studies [32] have shown that there cannot exist a double auction mechanism that possesses all of these properties simultaneously. In [33], new auction-based local energy market models that consider user preferences and willingness to pay for heterogeneous energy qualities were proposed. Existing and newly developed auction-based clearing algorithms were compared to identify one that satisfies pre-defined key characteristics, such as user preferences, willingness to pay, local coverage, individual rationality and computational traceability. These studies demonstrate the importance of carefully considering and evaluating different auction mechanisms in energy trading to achieve optimal performance in terms of efficiency, fairness and SW. Our previous study [10] compared auction mechanisms based on SW and energy traded, but it had limitations such as a small dataset, no bidding strategies, low SW and high computational time. Our proposed algorithm overcomes the limitations presented in the literature by introducing new features such as preference parameters, random and penny auction bidding strategies and peak and non-peak hour tariff distribution.

### Objective of this Study

From a comprehensive review of existing methodologies, a glaring gap emerges: that is the absence of an algorithm capable of dynamically changing the type of auction mechanism based on energy supply and demand intervals. Furthermore, while various auction mechanisms have been previously explored, the majority of studies have been confined to using random bidding strategies. These strategies, although popular, restrict adaptability and user engagement. The nature of random bidding fails to provide users with the flexibility to adjust their bids based on prior unsuitable bids, limiting the potential for efficient and effective P2P energy trading. Table 1 shows a comparison table of our proposed work with related work.

**Table 1:** Comparison of related work in auction mechanisms

Feature/study	[26]	[27]	[28]	[29]	[30]	[33]	[10]	Proposed work
Types of DA	4	3	4	2	2	1	4	4
User preferences	$\times$	$\checkmark$	$\times$	$\checkmark$	$\times$	$\checkmark$	$\times$	$\checkmark$
Selection of DA	$\times$	$\times$	$\times$	$\checkmark$	$\times$	$\times$	$\checkmark$	$\checkmark$
Bidding strategies	$\checkmark$	$\checkmark$	$\times$	$\checkmark$	$\times$	$\checkmark$	$\times$	$\checkmark$
Maximizes SW	$\times$	$\checkmark$	$\checkmark$	$\times$	$\checkmark$	$\checkmark$	$\checkmark$	$\checkmark$
Computation time	$\checkmark$	$\times$	$\times$	$\times$	$\times$	$\checkmark$	$\times$	$\checkmark$
MG to MG trading	$\times$	$\times$	$\times$	$\times$	$\times$	$\times$	$\times$	$\checkmark$
Time of usage	$\times$	$\checkmark$	$\checkmark$	$\times$	$\times$	$\times$	$\checkmark$	$\checkmark$

The proposed work introduces an innovative approach to P2P energy trading by integrating four distinct double auction mechanisms: Average Mechanism, McAfee Mechanism, Trade Reduction Mechanism and the VCG Mechanism. While double auction mechanisms have been explored in the energy trading domain, the novelty lies in the comprehensive comparative study of these mechanisms within various types of MGs - prosumer-centric, consumer-centric, EV-centric, equal distribution and no EV-based MGs. Additionally, the research goes beyond the typical application of these mechanisms by introducing a sophisticated algorithm that accommodates heterogeneous user preferences and incorporates time-specific tariffs (peak and non-peak hours) alongside two unique bidding strategies, random and penny auctions. This layered, flexible design system adeptly handles diverse MGs, making it novel. The following are the objectives of the proposed work:

1. A comparative study of four types of double auction mechanisms - namely, Average Mechanism, McAfee Mechanism, Trade Reduction Mechanism and VCG Mechanism - in the context of P2P energy trading. The study examines the properties of each mechanism - Individual rationality, Balanced budget, Truthfulness, Economic efficiency and Computational efficiency.
2. Proposed a novel P2P energy trading algorithm that incorporates all four auction mechanisms, along with bidding strategies, preference parameters and time of usage tariffs (peak and non-peak hours) to improve the efficiency and effectiveness of energy trading. This algorithm addresses the challenge of heterogeneous user preferences by allowing users to express their willingness to choose the most suitable DA and their preferences for specific time periods. Furthermore, the algorithm incorporates two different bidding strategies, random and penny auctions, which allow for greater flexibility and choice for prosumers.
3. Conducted a simulation study to evaluate the performance of the proposed algorithm on various types of MGs - prosumer-centric, consumer-centric, electric vehicle (EV) centric, equal distribution and no EV-based MGs. Additionally, we evaluate MG to MG energy trading to complete energy demand, providing valuable insights into the potential scalability of P2P energy trading.

These objectives fill the recognized gap by devising an algorithm that not only incorporates the four double auction mechanisms but also keenly addresses user preferences and introduces new bidding strategies. It allows users the flexibility to express their willingness to choose the most suitable DA and their time-specific preferences, setting it apart from other studies. Additionally, the extensive simulation study across various MG types, including MG to MG energy trading, illuminates the scalable potential of P2P energy trading, providing a more comprehensive perspective than previous studies.

The remaining sections of the paper are organized as follows. Section “Double Auction Mechanisms” provides an overview of the properties and types of double auction mechanisms. Section “Proposed P2P Energy Trading Framework” presents our proposed P2P energy trading framework, which incorporates bidding strategies, preference parameters and time of usage tariffs to improve the efficiency and effectiveness of energy trading. In Section “Case Study”, we conduct a case study to evaluate the performance of the proposed algorithm on various types of MGs. Finally, concluding remarks are drawn in Section “Conclusions and Future Work”.

## Double Auction Mechanisms

The term “auction” refers to a broad range of trading processes that establish a price for certain items, with the aim of conducting a competitive bidding process. DA mechanisms enable multiple consumers and prosumers to trade energy simultaneously within a single time interval. Let $C = {c_1, c_2, ..., c_n}$ represent the set of all consumers in the MG. Each consumer is denoted by $C_i$, where *i* is the index of the consumer and it ranges from 1 to *n*, indicating there are *n* consumers. Similarly, let $P = {p_1, p_2, ..., p_m}$ represent the set of all prosumers in the MG. Each prosumer is denoted by $p_j$, where *j* is the index of the consumer and it ranges from 1 to *m*, indicating there are *m* consumers. For every discrete time slot $t_i$, representing a specific trading interval, $D_{c_i}(t_i)$ signifies the energy demand of consumer $c_i$, while $S_{p_j}(t_i)$ indicates the surplus energy that prosumer $p_j$. The prosumer sets an ask price $p_{p_j}(t_i)$ for selling their surplus energy and on the other end, the consumer offers a bid price, represented by $b_{c_i}(t_i)$, for buying the energy demand at time slot $t_i$.

1 $$\begin{aligned} \text {Max} \sum \limits _{c_i \in C}\sum \limits _{t_i} (b_{c_i}(t_i) - p_{p_j}(t_i))x_{c_i, p_j}(t_i) \end{aligned}$$

The above equation represents the objective function for the double auction mechanism within the MG. The goal of this linear program is to maximize the total welfare of the MG. $x_{c_i, p_j}(t_i)$ is a binary decision variable that denotes whether a trade is successfully conducted between consumer $c_i$ and prosumer $p_j$, where a value of 1 indicates a successful trade and 0 denotes the absence of a trade. The expression $(b_{c_i}(t_i) - p_{p_j}(t_i))x_{c_i, p_j}(t_i)$ computes the difference between the bid price of a consumer and the ask price of a prosumer at time slot $(t_i)$. The double summation $\sum \limits _{c_i \in C}\sum \limits _{t_i}$ ensures that this computation is done for every consumer, for every time slot, thus considering all possible trades. This objective function maximizes the total welfare of the MG by summing the surpluses of all the players who participate in the trades. The goal of the double auction mechanism is to find a set of trades between consumers and prosumers that maximizes the SW of the MG.

In P2P energy trading, a community aggregator or auctioneer gathers data from both participants: consumers and prosumers. The auctioneer organizes the prices, determining the trading price required to balance the market [34]. Serving as a network operator, the auctioneer facilitates connections between peers and MGs (MGs). Its role includes overseeing P2P energy trades, ensuring these transactions don’t undermine the integrity of the distribution network. Furthermore, the auctioneer is tasked with ensuring that supply and demand within the MGs are in equilibrium, maintaining stability in the distribution network. However, centralized double auctions present multiple challenges: (a) the failure of the auctioneer can halt the entire trading algorithm, (b) a group of peers can merge with the auctioneer to create a biased price for trading and (c) the auctioneer needs to consider the geographical distance between buyer and seller to minimize distribution losses, creating dependency and centralizing the system again. As the objective of the proposed model is to make P2P energy trading decentralized, the trading algorithm fulfils all the objectives of the auctioneer and is used to match, pair and handle transactions.

**Fig. 1:**
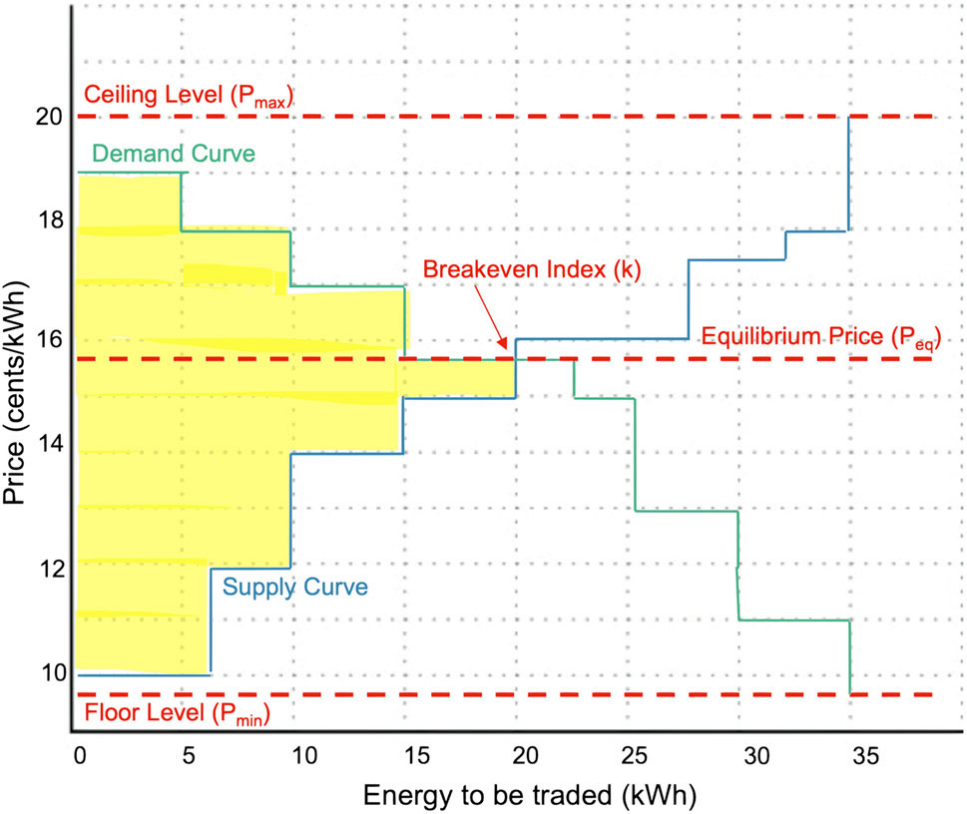
A demonstration of how equilibrium price and breakeven index is calculated in double auction

### Natural Ordering and Breakeven Index

The sensitivity of the buying and selling price difference is bounded by the floor and ceiling prices, which are represented by the minimum price $P_{\min }$ and the maximum price $P_{\max }$, respectively. The floor price, denoted as $P_{\min }$, is the minimum acceptable price at which a trade can occur in the system. It acts as a safety price, ensuring that prices do not fall below a level that might be disadvantageous to sellers, leading them to incur losses or discourage participation. On the other side, the ceiling price, represented by $P_{\max }$, is the maximum allowable price for a trade. This upper limit ensures that buyers are not overcharged or exploited due to high demand or other market conditions. These boundary prices are determined by the minimum feed-in tariff that the Distribution System Operator (DSO) pays to an independent prosumer and the maximum tariff that the DSO charges consumers [10]. Depending on the load curve, these floor and ceiling prices can remain consistent throughout the day or vary during peak and non-peak hours. It is important to note that the bids and offers submitted by prosumers and consumers, as well as the trading price established by the auction mechanism, should always remain within the bounds of $P_{\min }$ and $P_{\max }$. If a prosumer offers a price higher than $P_{\max }$, consumers would prefer to purchase energy from the grid, resulting in no transaction. Conversely, a prosumer’s offer exceeds $P_{\min }$ because it represents the price the prosumer intends to receive in exchange for trading excess energy in the P2P market. The situation is identical when a consumer places a bid.

After participants submit their respective bids and asking prices, the Equilibrium price $P_{Eq}$ is derived using Natural Ordering and the Breakeven Index methodology. In the Natural Ordering process, consumer bids are arranged in descending order based on price, forming the demand curve when plotted on a graph. Conversely, prosumer offers are organized in ascending order by their prices and, when charted, result in the supply curve. The intersection of these two curves on the same graph yields the Breakeven point or the Breakeven Index, as illustrated in Fig. 1. This index represents the exact point where supply meets demand. The point of intersection also determines the $'k'$ users selected for trading. In scenarios where multiple buyers or sellers submit identical bids, priority is granted to those with the most energy available for trade. This prioritization ensures a maximization of energy trade volume, thus enhancing SW. Geometrically, the region enclosed by the supply and demand curves alongside the price axis symbolizes the total SW, depicted by the yellow segment in Fig. 1. An efficient market equilibrium is realized when this SW is maximized. By leveraging Natural Ordering and the Breakeven Index to determine the $P_{Eq}$, the proposed P2P energy trading model guarantees a trading price that is both equitable and indicative of supply-demand dynamics.

### Properties of Auction Mechanisms

The Myerson-Satterthwaite theorem [32] establishes that no practical mechanism can exhibit all properties simultaneously despite an ideal auction mechanism displaying the first four properties listed above. Therefore, the order in which these four elements are prioritized depends on the specifics of the local market and the MG type.

#### Individual Rationality(IR)

The principle of Individual rationality (IR) requires that no participant should be worse off by participating in a DA. This means that the trading price must be greater than or equal to the asking price at breakeven index $'k'$ and less than or equal to the bidding price at breakeven index $'k'$, i.e., $BP_k \ge TP_k \ge AP_k$. $BP_k$. In other words, the price represents what a consumer bids in the auction to purchase energy. Furthermore, every peer participating in the auction should achieve a non-negative utility. This ensures that prosumers are not compelled to decrease their asking price when selling energy to the grid and consumers are not burdened to pay more than the grid price upon winning the auction [35]. Satisfying the IR ensures that $U_{n}(\chi _{n}) \ge 0$ for each peer. This implies that every auction market participant attains a non-negative utility during the auction process.

#### Balanced Budget(BB)

The Balanced Budget (BB) property is a crucial aspect of mechanism design. It guarantees the financial sustainability of the mechanism, ensuring that the auctioneer or the platform neither profits nor incurs a loss in terms of utility. There exist two types of BB: strong and weak. In a strong BB, the auctioneer or platform maintains a neutral utility, neither gaining nor losing. Conversely, in a weak BB, while the auctioneer or platform is protected from losses, they might accrue gains. Given that our proposed platform serves as a trading platform for energy, it upholds a strong BB. This characteristic ensures the platform’s financial stability while fostering a transparent and efficient market for energy trading.

#### Truthfulness or Incentive Compatibility (IC)

Truthfulness, also known as Incentive Compatibility (IC), is a property in auction mechanism design. It incentivizes all participants to report their genuine values, eschewing any strategic deception. There are two forms of IC: strong and weak. In the strong notion of IC, participants aren’t necessarily required to reveal their true values to everyone since it’s considered a dominant strategy. In contrast, the weak IC operates under the Nash equilibrium, ensuring all participants remain truthful by revealing their genuine values. Achieving truthfulness within the auction can be intricate, but it’s feasible by meticulously monitoring transactions. An auction strategy that satisfies the IC property guarantees the following relationship: $U_{n}(\chi _{n}) \ge U_{n}(\kappa _{n})$, where $\chi _{n}$ and $\kappa _{n}$ represent the true and false bids of peer *n*, respectively. This property reinforces the idea that participants in the auction market reap the most benefits only when they declare their authentic bids.

**Table 2:** Types of double auction mechanism with their properties

Property name	Name of the mechanism			
	*Average*	*VCG*	*Trade reduction*	*McAfee*
Individual rationality	Yes	Yes	Yes	Yes
Balanced budget	Yes	No	No	No
Truthfulness	No	Yes	Yes	Yes
Economic efficiency	Yes	Yes	No	No
Trading price	Mid-market	Table 3	BP & SP at k	1. Mid-market
				2. BP & SP at k
Players	$C_k$ & $P_k$	$C_k$ & $P_k$	$C_{k-1}$ & $P_{k-1}$	1. $C_k$ & $P_k$
				$C_{k-1}$ & $P_{k-1}$

#### Economic Efficiency (EE)

Economic Efficiency (EE) is a fundamental property in auction theory, aiming to maximize social welfare (SW), which represents the combined utility of all participants. In energy auctions, EE is achieved when the trading price matches the equilibrium price—where the supply and demand curves intersect—resulting in optimal SW. The core objective of an auction mechanism is to enhance SW as an indicator of economic efficiency. Consequently, a trading platform should consistently strive to maximize SW to ensure the best resource allocation.

To compute SW in a double auction, it’s essential to first establish the buying and selling prices. After determining these prices, the surpluses for both the buyer and seller can be calculated. The sum of these surpluses gives the total SW. Mathematically, *SW*, represents the net benefits accumulated by all entities in the double auction and it can be articulated as:

2 $$\begin{aligned} SW = \sum \limits _{c_i \in C}\sum \limits _{p_j \in P} (v_{c_i} - c_{p_j})x_{c_i, p_j} \end{aligned}$$

In this equation, *C* is the collection of all buyers with a total of *n* consumers. Similarly, *P* symbolizes all the sellers in the market, represented by *m* prosumers. The term $v_{c_i}$ denotes the valuation of buyer *i*, illustrating their willingness to pay or their perceived value of the energy. Conversely, $c_{p_j}$ denotes the valuation of seller *j*, which effectively is the lowest price the seller is willing to accept for their energy offering. $x_{c_i, p_j}(t_i)$ is a binary decision variable that denotes whether a trade is successfully conducted between consumer $c_i$ and prosumer $p_j$, where a value of 1 indicates a successful trade and 0 denotes the absence of a trade. This cumulative sum yields the total SW, which, when maximized, signifies a state of Nash equilibrium in the double auction paradigm. At this equilibrium, the energy quantity desired by consumers matches what the prosumers offer, generating transactions at a balanced equilibrium price.

#### Computational Efficiency (CE)

Computational Efficiency (CE) denotes the capability of an auction mechanism to operate within a computationally feasible timeframe. Specifically, a DA is deemed computationally efficient if it can determine the winning peer set, establish a matching rule and finalize the clearing payment in a timely manner. In essence, the auction shouldn’t incur excessive execution times and its computational demands must align with the resources of the available hardware and software. This characteristic becomes particularly critical in large-scale energy markets with numerous participants, where swift and efficient auction execution is paramount. Hence, computational efficiency remains a essential property for double auction mechanisms.

### Types of Auction Mechanisms

Types of DA mechanism with their properties, trading price and players are illustrated in Table 2.

#### Average Mechanism

The trading price $TP_{Avg}^{c,p}$ in the average mechanism is the mid-market value. Specifically, it is the arithmetic mean of the bid price from the consumer, $BP_{Avg}^{c}$ and the ask price from the prosumer, $AP_{Avg}^{p}$. By taking the average of these two values, the mechanism ensures that the trading price is a fair representation of both the buyer’s willingness to pay and the seller’s minimum acceptable price. The resulting price strikes a balance between the two, making the trade mutually beneficial.

3 $$\begin{aligned} TP_{Avg}^{c,p}= \frac{BP_{Avg}^{c} + AP_{Avg}^{p}}{2} \end{aligned}$$

In the average mechanism [22], IR is ensured. By ordering bids and asks, it ensures that no participant will engage in trade below their reservation price. The mechanism also satisfies the BB criterion, given that the aggregate payment from the winners matches the collective cost lost by the losers. EE is also achieved since the energy is allocated to the $'k'$ players who value it the most, optimizing SW. However, the average mechanism does not adhere to IC. Participants might gain an advantage by submitting bids or asks that misrepresent their actual valuations. Specifically, consumers could bid at lower prices to secure more favorable terms, while prosumers might inflate their asking prices to boost their earnings. Such strategic behaviors could usher in market inefficiencies and result in a less-than-optimal resource distribution. Haggi and Sun [36] elaborates on a multi-round double auction underpinned by an average pricing mechanism, highlighting its merits from both technical and computational perspectives.

#### Vickrey-Clarke-Groves(VCG) Mechanism

VCG mechanism [37] is a first-price sealed-bid mechanism designed for multiple players. It strives to optimize SW while ensuring truthfulness. In this mechanism, consumers submit sealed BP, while prosumers present sealed AP. These bid and ask prices are naturally ordered: bid prices are arranged in ascending order and ask prices in descending order. A trade occurs if $BP_k \ge AP_k$, where *k* is a predetermined constant in the range [0, 1] and the price is determined according to Table 1. The mechanism calculates the breakeven index *k* using natural ordering and trades occur between the first *k* consumers and the first *k* prosumers.

The VCG mechanism satisfies several important properties. It upholds IR since consumers pay less than their true value and prosumers receive more than their actual worth. The mechanism also ensures IC, as both consumers and prosumers have the incentive to reveal their true values. Additionally, it promotes EE by maximizing SW. However, the VCG mechanism falls short in achieving BB since the auctioneer does not derive any incentives from the trade. In the study by [24], the authors put forward a P2P energy trading framework. This model leans on bilateral agreements, incorporates the VCG mechanism and facilitates trades with the DSO. Nevertheless, it takes into account peer preferences, which can result in a reduced SW. Another research work [38] contrasts the pay-as-bid mechanism with the VCG mechanism in the electricity market. It harnesses both game theory and auction theory to suggest alternative mechanisms. Furthermore, [39] refines the VCG mechanism for electricity markets that employ a bid system for control reserves. Their work underscores the efficiency and IC of the VCG mechanism. However, they also recognize its potential to escalate costs for the market-maker, especially when participant coalitions are present. Table 3 below shows Possible cases to calculate $P_{Eq}$ for VCG.

**Table 3:** Possible cases to calculate equilibrium price for VCG

	$AP_{k+1} > BP_k$	$AP_{k+1} \le BP_k$
$BP_{k+1} \le AP_k$	$AP_k , BP_k$	$AP_k , AP_{k+1}$
$BP_{k+1} \ge AP_k$	$BP_{k+1} , BP_k$	$BP_{k+1} , AP_{k+1}$

#### Trade Reduction Mechanism

The Trade Reduction (TR) auction mechanism limits trades to just $k-1$ consumers and prosumers. This design helps prevent trade subsidization and ensures that participants bid truthfully. The TR mechanism, as described by [40], first involves calculating the natural ordering and breakeven index, much like the average mechanism. After that, the market institution picks $k-1$ consumers and prosumers to trade energy. The top $k-1$ prosumers sell their energy at a price of $AP_k$ while the first k-1 consumers pay the buying price $BP_k$

This TR mechanism upholds IR, meaning consumers end up paying less than their valuation, while prosumers receive more than theirs. It’s also designed to be IC because the chosen consumers and prosumers have no reason to change their bids — any changes wouldn’t impact the trading price. However, the mechanism doesn’t fully achieve a BB since the auctioneer remains with extra energy. Moreover, it doesn’t optimize EE. The reason is that the $k^{th}$ consumer and prosumer can’t join the TR auction, which leads to a less-than-ideal SW. And if we try to include this $k^{th}$ player, it would change the market prices and compromise the mechanism’s truthfulness.

#### McAfee Mechanism

The McAfee mechanism combines elements from both the Trade Reduction and Average mechanisms to optimize P2P energy trading. Initially, the mechanism determines the breakeven index and carries out natural ordering. Subsequently, it examines the trading price to decide whether the first $k^{th}$ consumers and prosumers should trade energy or if the trade reduction mechanism should be applied to $(k-1)^{th}$ players instead. While the McAfee mechanism is IR and truthful, it doesn’t achieve a BB or EE in the latter scenario. A study by [41] introduced a flocking-based McAfee mechanism and juxtaposed it with a centralized algorithm for P2P energy trading in neighborhoods. Their findings showed that the McAfee-based double auction algorithm surpassed the centralized approach in energy trading efficiency.

## Proposed P2P Energy Trading Framework

P2P energy trading in a microgrid involves various participants: consumers (*i*), prosumers (*j*), and EVs represented by *v*. In this model, $E_{ij}$ denotes the energy traded from prosumer *j* to consumer *i*, whereas $E_{vj}$ represents the energy traded from prosumer *j* to EV *v*. Each prosumer *j* has an energy production of $S_{p_j}$, while each consumer *i* and EV *v* have energy demands represented by $D_{c_i}$ and $D_{v}$ respectively. Sometimes, the microgrid might need to draw energy from the main grid, and this amount is indicated by $P_{grid}$. Lastly, the cost per unit of energy is represented by $C_{E}$.

The main objective is to minimize the overall cost of energy trading within the microgrid. This is calculated as the sum of the products of the energy traded and its respective cost, adjusted for any energy drawn from the main grid. The system is governed by constraints to ensure energy conservation at each prosumer, consumer, and EV, as well as non-negativity constraints to ensure no negative energy trading. Additionally, there might be times when the combined demand from consumers and EVs exceeds the supply from prosumers, necessitating drawing power from the main grid, represented by the grid energy requirement constraint.

4 $$\begin{aligned} \text {Minimize } \sum C_{E} \times (E_{ij} + E_{vj} - P_{grid}) \end{aligned}$$

5 $$\begin{aligned} s.t. S_{p_j} = \sum _{i} E_{ij} + \sum _{v} E_{vj} \quad \forall j \end{aligned}$$

6 $$\begin{aligned} D_{c_i} = \sum _{j} E_{ij} \quad \forall i \end{aligned}$$

7 $$\begin{aligned} D_{v} = \sum _{j} E_{vj} \quad \forall v \end{aligned}$$

8 $$\begin{aligned} E_{ij} \ge 0, \quad E_{vj} \ge 0 \quad \forall i, j, v \end{aligned}$$

9 $$\begin{aligned} P_{grid} \ge \left( \sum _{i} D_{c_i} + \sum _{v} D_{v} \right) - \sum _{j} S_{p_j} \end{aligned}$$

**Fig. 2:**
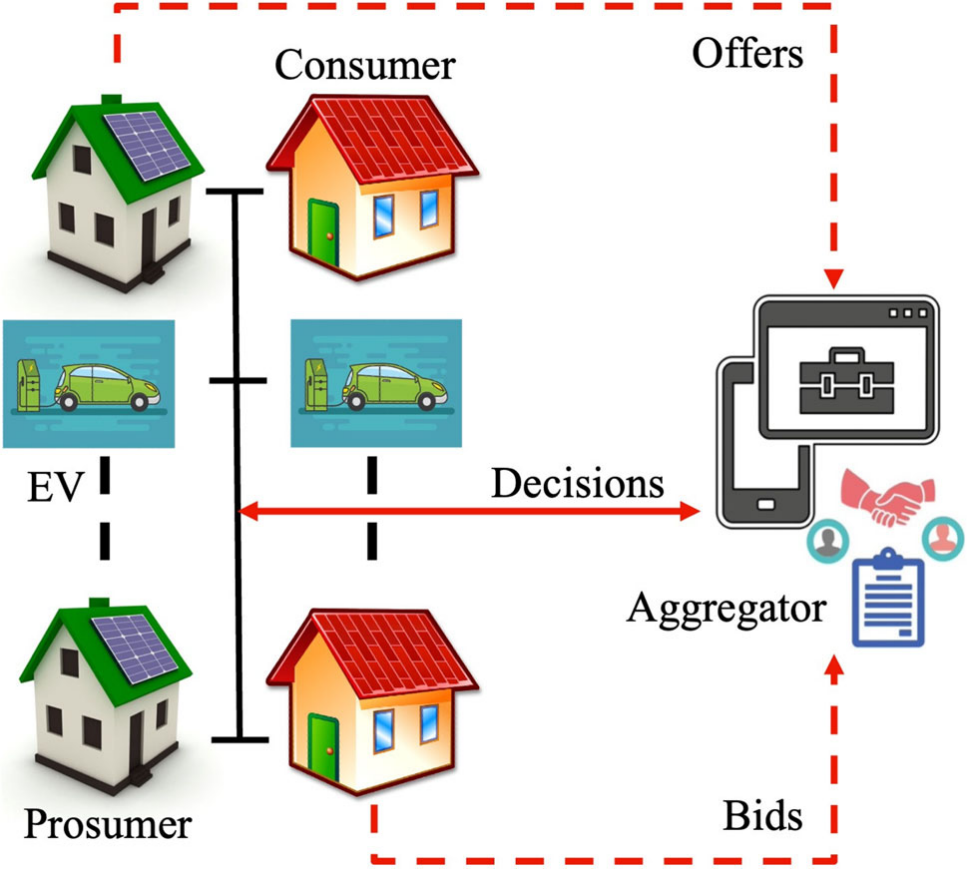
System model of a microgrid with consumers, prosumers, EV and aggregator

**Fig. 3:**
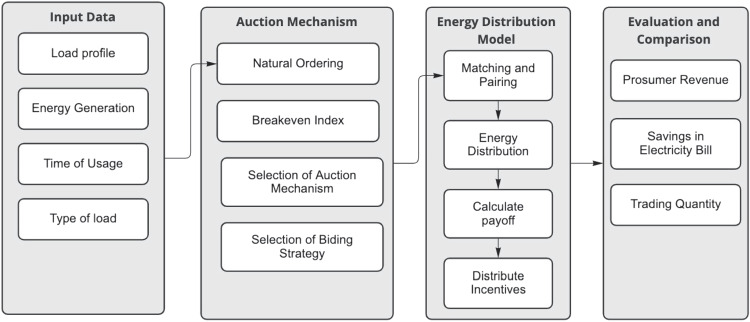
Simulation setup for DSA mechanism algorithm

The concept of a MG has gained significant attention because of its capacity to distribute and regulate electricity in a localized version of the main distribution network. An MG is essentially a compact power system comprising multiple distributed generators, usually situated close to the load [42]. In this paper, our focus is on a representative community MG system. This system encompasses consumers, prosumers, an aggregator and EV charging points. All these entities are interconnected through the community aggregator, as illustrated in Fig. 2. The proposed P2P energy trading framework is depicted in a comprehensive four-step auction mechanism, as showcased in Fig. 3. Initially, during the Input Data Stage, peers relay their data to the trading platform. This is followed by the Auction Mechanism Stage where the most suitable auction type is determined and associated bidding strategies are introduced. The process progresses to the Energy Distribution Model Stage, where peers are aptly paired, facilitating the energy distribution to consumers. The procedure culminates in the Evaluation and Comparison Stage, wherein the framework’s efficacy is critically assessed and benchmarked against findings from prior studies.

### Input Data

In the Input Data phase of the P2P energy trading framework, the community aggregator initiates the DA process for all peers within the MG. This is followed by a registration window where peers need to sign up for participation, completing their registration half an hour prior to the predetermined trading time slot, ${t_i}$. Even though trading intervals can range from 5 to 30 minutes, this study uniformly adopts a 30-minute slot. Prosumers, identified as $P = {p_1, p_2, ..., p_m}$, assess their energy patterns and then, based on their energy dynamics, decide whether to engage in the P2P market as consumers or prosumers. Within the DA market, while consumers provide their bid price (*BP*) showcasing the upper limit they’re willing to pay, prosumers present their ask price (*AP*), denoting the minimum they expect for their surplus energy. Taking the DA a step further, this study explores a mechanism where one prosumer has the capability to trade with multiple consumers and vice-versa. Trades are executed when the condition $BP \ge AP$ holds true, with the community aggregator finalizing the exact trade price. Conversely, if $BP < AP$, no trade is executed. The stage concludes with the auctioneer launching the bidding process, wherein participants, both consumers and prosumers, submit crucial information including ask price, selling price, location, energy demand, and surplus energy availability for the respective time slot.

#### Preference Parameters

Preference parameters play an essential role in shaping the energy trading decisions among peers in the P2P market. These parameters reflect a player’s interest or disinterest to engage in trades with specific peer based on various factors. For instance, a consumer might be inclined to trade with a nearby prosumer or with one who consistently has surplus energy available at a required time. On the other side, a prosumer might lean towards a consumer who offers a higher price or has previously exhibited a trustworthy payment record. By considering these preferences, participants enhance their likelihood of being paired with their desired trading associates, optimizing their trade and outcomes. This not only personalizes the peer preference but also enhances the overall efficiency and effectiveness of the DA system.

To put these preferences mathematically, the preference of a consumer *c* towards trading a prosumer*p*, is represented as $\alpha _{c,p}$. Conversely, a prosumer’s *p* inclination to trade with a consumer *c* is symbolized by $\alpha _{p,c}$. For clarity, if consumer *c* earmarks a value of $\alpha _{c,p}$ = 1 for a prosumer *p*, it signifies *c*’s readiness to procure energy from *p*. In a similar way, when prosumer *p* designates $\alpha _{p,c}$ = 1 for a consumer *c*, it indicates *p* willingness to vend energy to *c*.

#### Time-of-usage Tariff

In the Time of Usage (ToU) tariff [43], which is based on energy demand for an extended time, like an hour, daytime and nighttime. Information regarding ToU is provided in advance to the users in the MG and stays constant for a more extended time. Under the ToU scheme, the day is divided into peak, shoulder peak and off-peak periods. The total energy bill or cost $\mathbb {B}_{n}^{\textrm{TOU}}$ for a user under the ToU tariff over a specified period can be calculated by [44]:

10 $$\begin{aligned} \mathbb {B}_{n}^{\textrm{TOU}}=\sum _{t=1}^{T} K^{t} \times Q_{load}(t) \end{aligned}$$

where $K^{t}$ represents the ToU tariff rate at time interval *t*, depending on on whether the time *t* falls under peak, shoulder, or off-peak periods, as elaborated in the second Eq. 5. $Q_{load}(t)$ is the total demand of the user in time interval *t*:

11 $$\begin{aligned} K^{t}= {\left\{ \begin{array}{ll}K^{\text{ peak } } &{} \text{ if } t \in t^{\text{ peak } } \\ K^{\text{ shoulder } } &{} \text{ if } t \in t^{\text{ shoulder } } \\ K^{\text{ off-peak } } &{} \text{ if } t \in t^{\text{ off-peak } }\end{array}\right. } \end{aligned}$$

where $K^{\text{ peak }}$ is the ToU tariff rate during peak hours, $K^{\text{ shoulder }}$ is the ToU tariff rate during shoulder hours, which typically are the times between peak and off-peak periods, and $K^{\text{ off-peak } }$ is the ToU tariff rate during off-peak hours. The proposed trading algorithm divides the entire day into peak and non-peak hours. In this study, the peak-hour floor and ceiling price is assumed to be $P_{\min } = 0.09h$ €/kWh and $P_{\max } = 0.20$ €/kWh, respectively. Similarly, for non-peak hours the floor and ceiling prices assumed are $P_{\min } = 0.09$ €/kWh and $P_{\max } = 0.15$ €/kWh, respectively.

The application of ToU tariffs have implications on DA P2P energy trading. Firstly, ToU tariffs can affect the demand and supply of energy by incentivizing consumers to shift their energy consumption to off-peak hours, which can affect the amount and timing of energy available for trading, impacting auction prices and SW. Secondly, ToU tariffs can lead to more price volatility, creating uncertainty for buyers and sellers, which may impact their willingness to participate in the auction. Finally, ToU tariffs can create incentives for strategic behaviour by buyers and sellers, resulting in inefficiencies in the auction and potentially reducing SW.

### Auction Mechanism and Bidding Strategies

The second stage of this mechanism is auction mechanism and bidding strategies and initiates with: natural ordering and the breakeven index. In natural ordering, all bids from consumers are sorted in descending order for their price and all offers from prosumers are sorted in ascending order with respect to their prices. The sorted bids are plotted on the graph and form a demand curve. Similarly., the sorted ask price, when plotted on the graph, makes a supply curve. Both the supply and demand curve, when plotted on the same graph, provides a breakeven point, also known as the Breakeven index, as shown in Fig. 1. The point where the supply curve and demand curve meets gives us the number of ’k’ user to be considered for trading, as explained in Section “NaturalOrdering and Breakeven Index”. The peers inside the yellow shaded area in every shot get selected for trading and others are given chance again in the next shot. This process keeps on going until unless all the peers have fulfilled their energy demand or no other prosumer is left to provide its excess energy. The initial bidding, or the “first shot,” is initiated randomly within our algorithm, constrained by the floor and ceiling prices. Once this is accomplished, remaining consumers, still in need of energy, and prosumers, with surplus energy on hand, re-enter the bidding process. After the initial bid, the strategy pivots to the Pay-per-bid auction model, a variation of the penny auction. The motive behind using penny auction over random bidding lies in the understanding gained from the initial round: peers recognize the necessity to tweak their bids, either incrementally or decrementally, to align with the breakeven index, ensuring their inclusion in subsequent shots/rounds. This strategic modification not only accelerates the selection process but also significantly decreases the number of shots and transactions, promoting efficiency. The penny auction model [45], as explored in this study, is characterized by bids oscillating by a precise one cent, either upwards or downwards. This approach, influenced by platforms like Quibids which allow increments from €0.01 to €0.15 [46], ensures that the winning bid’s value benefits all participants, solidifying its effectiveness for the described application.

A modified penny auction can be illustrated as a tuple $(N, \chi _{n}, P_{\min }, \epsilon )$. In this tuple, *N* is the number of consumers or prosumers participating in the auction, $\chi _{n}$ is the true value, $P_{\min }$ is the minimum bid cost(floor level) and $\epsilon$ is the fixed increment price of the bid. At any time interval $t_i$, bids may be placed during the auction, which begins with a set price, $P_{\min } < \chi _{n}$. An optimal strategy profile for this game, or one from which no players would deviate, is known as a Nash equilibrium. This is true as long as players think that all other players are operating symmetrically. In a penny auction, every Nash equilibrium corresponds to a different selling price *AP*. However, the increment or the *AP* cannot be more than $P_{\max }$ i.e. ceiling level. Revenue, denoted by *R*, in this setup, is determined by the aggregate of bids placed and the final selling price and can be mathematically expressed as:

12 $$\begin{aligned} AP \le \max {[(\chi _{n}-\epsilon )+\epsilon * (N-1), P_{\min }+\epsilon * N]} \end{aligned}$$

13 $$\begin{aligned} R \le \frac{AP}{\epsilon } + AP \end{aligned}$$

#### Energy Distribution Model

The process of energy distribution is arranged to ensure the optimal pairing of consumers and prosumers, taking into account their individual demands, supplies, and preferences. Bids placed by the participants are first bifurcated into supply and demand categories. Supply prices are arranged in ascending order, primarily based on their sell limits, with the highest supply quantity given preference. Similarly, demand prices are sequenced by their buy limits, again favoring the highest demand quantity. Essentially, prosumers (those who produce energy) begin the trading process with consumers willing to pay the most, or who have placed the highest bids. As these highest bidders are catered to, the process then shifts to the next highest bidders, ensuring a hierarchical trading sequence. This method is called matching and pairing. Upon a successful match between supply and demand bids, these bids are removed from their respective stacks. The trading algorithm then assesses the price and quantity specifications of the subsequent pair of bids. In instances where multiple participants exhibit congruent bids and offers, priority is accorded to the one showcasing a greater energy deficit or an abundance of energy for trading. Should any consumers or prosumers remain unmatched for the current bid, they are earmarked for potential consideration in the ensuing shot but within the same time frame. After the trading shots, the system calculates the payoff for each peer. This is derived from the difference between the prices at which they bought or sold energy and the predetermined floor or ceiling prices, or any other benchmark prices used in the system.

14 $$\begin{aligned} {\begin{matrix} {U_{pros}(t_i)} = \alpha _{p,c} \log _2[1 + Qty_{Grid} + Qty_{p2p}] \\ + P_{fit} Qty_{Grid} +TP Qty_{p2p} \end{matrix}} \end{aligned}$$

15 $$\begin{aligned} {\begin{matrix} {U_{cons}(t_i)} = \alpha _{c,p} \log _2[1 + Qty_{Grid} + Qty_{p2p}] \\ - P_{grid} Qty_{Grid} - TP Qty_{p2p} \end{matrix}} \end{aligned}$$

The utility function of the prosumer at a specific time interval is denoted by ${U_{pros}(t_i)}$. Conversely, ${U_{cons}(t_i)}$ represents the utility function of the consumer during the same interval. In these equations, ${\alpha _{p,c}}$ reflects the preference of a prosumer to trade with a consumer, whereas ${\alpha _{c,p}}$ signifies the preference of a consumer to engage in trade with a prosumer. The quantity of energy transacted with the central grid is expressed as ${Qty_{Grid}}$, while the amount of energy exchanged directly in peer-to-peer trading is represented by ${Qty_{p2p}}$. The earnings of a prosumer from redirecting energy to the grid is determined by the feed-in tariff, ${P_{fit}}$, and the earnings from direct peer-to-peer transactions are established by ${P_{p2p}}$. On the other side, ${P_{grid}}$ stands for the rate at which consumers acquire energy directly from the grid.

**Fig. 4:**
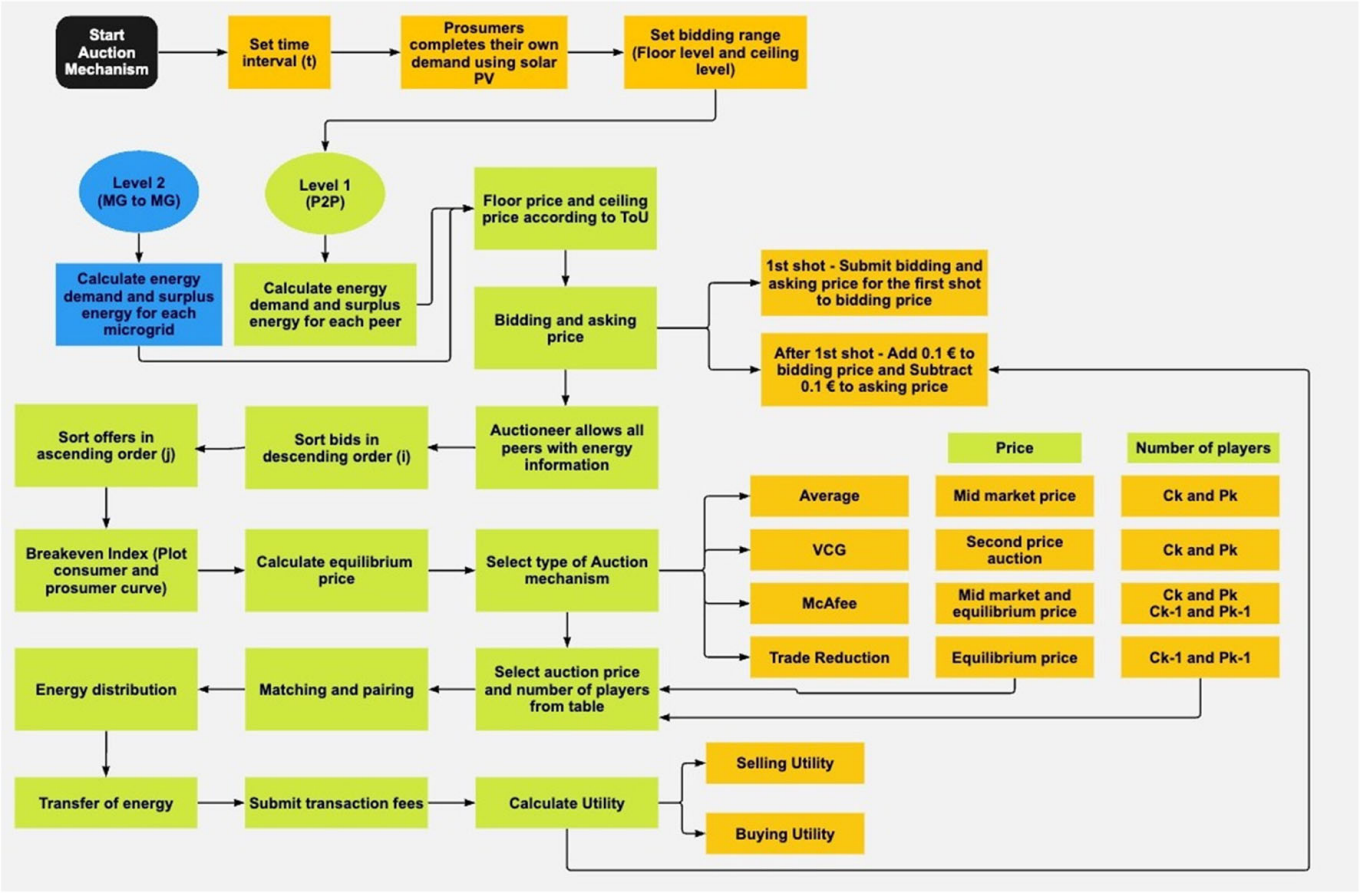
Visualizing the flowchart for P2P energy trading algorithm for all double auction mechanism

The entire process is shown in the form of a flowchart in Fig. 4. The flowchart provides a visual representation of the complex algorithm and makes it easier for users to understand the various steps involved in the P2P energy trading process. The flowchart depicts the P2P energy trading algorithm using a double auction mechanism. The orange colour options represent the user choices that are available to select in the algorithm, while the green colour represents the actual flowchart of the algorithm for P2P energy trading. The flowchart outlines the steps involved in the P2P energy trading process, including bid submission, bid matching and price determination. The blue colour tabs (level 2) represent the subsequent steps that will be taken once the green-coloured algorithm (level 1) is completed, including the calculation of incentives for prosumers and consumers.

**Fig. 5:**
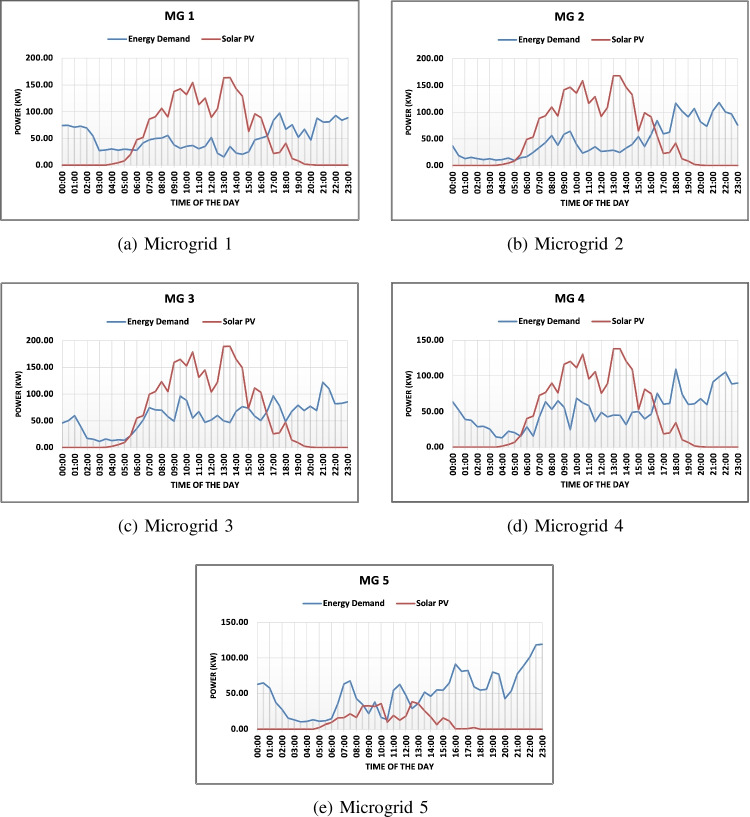
Local net generation and consumption with different type of MGs

## Case Study

In the following section, we present a detailed simulation of our proposed DA trading algorithm, tailored for P2P energy trading within the context of the IEEE European Low Voltage Test Feeder [47]. The dataset used for this simulation spans 24 hours; however, generation data is exclusive to daylight hours, given that it stems from solar rooftops. This dataset has been compartmentalized into five distinct MG scenarios, each characterized by varying degrees of participation from consumers, prosumers, and EV. The five MG scenarios included an equal distribution scenario, a prosumer-centric scenario, a consumer-centric scenario, an EV-centric scenario and a scenario without any EV charging stations. As illustrated in Fig. 5, the energy generation and demand patterns for each MG have been meticulously analyzed over a continuous 24-hour window. Prosumers, in this model, initially cater to their personal energy needs. Any surplus energy—determined by the discrepancy between distributed solar PV generation and domestic energy consumption—is then channeled to other users within the network. It’s worth noting that prosumers may transition into a consumer role during specific time intervals, denoted as *T*, especially when their energy production falls short of their energy requirements. Table 4 delves into specifics, shedding light on the count of consumers, prosumers, and EV charging stations present in each MG. Moreover, Fig. 5 vividly portrays the energy demand against photovoltaic (PV) generation data, across all five MG scenarios.

To ensure the accuracy and reliability of our algorithm, extensive tests and simulations were conducted using Python. We opted for Python over Matlab primarily due to its superior computational efficiency. To illustrate, Python’s processing time for the inaugural run stood at a mere 6 seconds for each MG, which then reduced to 2 seconds for subsequent time intervals. This agility positions it as an ideal tool for real-time trading endeavors. Conversely, when executed on Matlab, the same algorithm demanded a considerably longer duration, peaking at 3 minutes for every individual MG.

In this case study, the proposed trading algorithm was compared against four different auction mechanisms, with bidding strategies assessed using several performance metrics. We evaluated the performance of the proposed mechanism from various perspectives, including the total revenue generated, the average utility for both consumers and prosumers, and the number of rejected bids. The results of this case study revealed that the proposed trading algorithm consistently outperformed the other auction mechanisms, underscoring its potential in facilitating P2P energy trading within MGs. To thoroughly assess the performance of the proposed P2P energy trading algorithm, we examined it under four distinct scenarios:

1. Scenario 1: Considering only Penny auction bidding strategy
2. Scenario 2: Considering only Time of Usage Tariff
3. Scenario 3: Considering Penny auction bidding strategy and Time of Usage Tariff
4. Scenario 4: A basic scenario where there is a fixed price for all day and bidding is done randomly for each shot

**Table 4:** Number of peers distributed in MGs

		Prosumers	Consumers	EV
MG 1	Equal distribution	10	10	3
MG 2	Prosumer centric	15	5	3
MG 3	Consumer centric	5	15	3
MG 4	No EV’s	10	10	0
MG 5	EV centric	5	5	6

Table 5 summarizes the various scenarios that are studied for different MGs. The highlighted value indicates the best possible scenario for each DA mechanism. Several results can be drawn from Table 3. The table shows the average energy traded in kWh and the SW in €for each scenario using four different auction mechanisms: Average, Trade Reduction, VCG and McAfee. The highest average energy traded for MG 1 was seen in Scenario 1 using VCG, which yielded 317 kWh, whereas the lowest was seen in Scenario 2 using McAfee, which yielded 112 kWh. For MG 2, the highest average energy traded was seen in Scenario 3 using VCG, which yielded 333 kWh, whereas the lowest was seen in Scenario 2 using McAfee, which yielded 107 kWh. In terms of SW, the highest value for MG 1 was seen in Scenario 1 using VCG, which yielded €53, while the lowest was seen in Scenario 2 using the Trade Reduction mechanism, which yielded €23. For MG 2, the highest SW was seen in Scenario 3 using the Average mechanism, which yielded €86, while the lowest was seen in Scenario 2 using McAfee, which yielded €24. Overall, the VCG mechanism performed well in terms of energy trading and SW in both MG scenarios. The Trade Reduction mechanism performed poorly in terms of both energy trading and SW.

**Table 5:** Median factor of frame size increase due to a keyframe injection

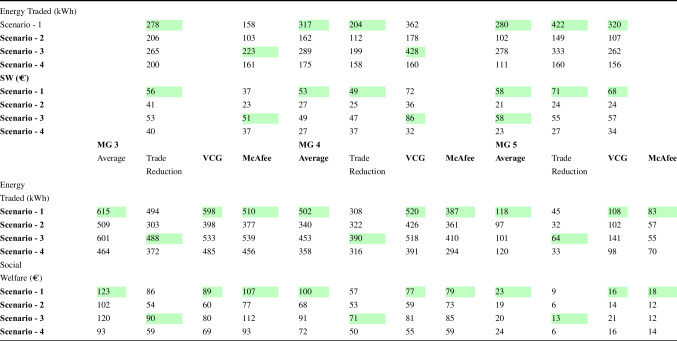	

**Fig. 6:**
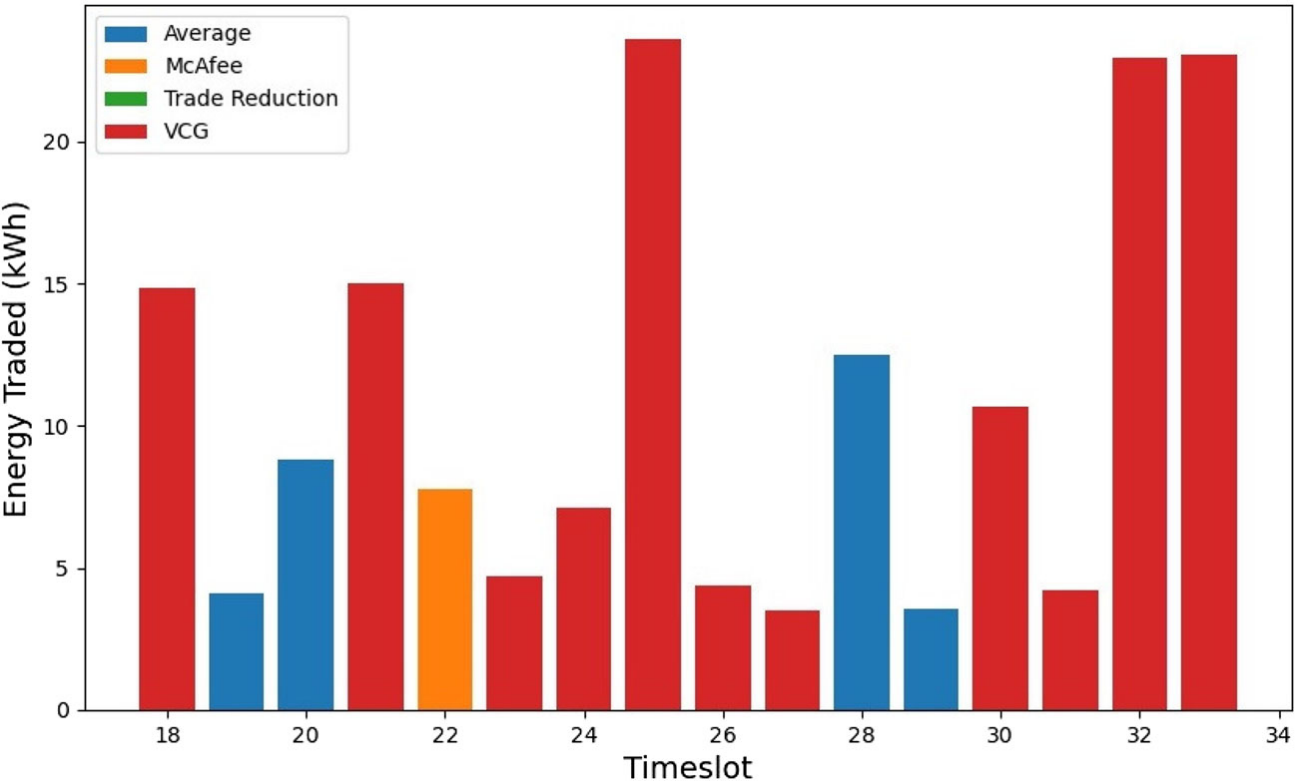
Selection of auction mechanism for each time slot based on the energy trading using the best possible scenario

In MG (MG) 3, which is a consumer-centric MG comprising three electric vehicle (EV) charging points, the auction mechanism once again resulted in the highest energy traded, amounting to 615 kWh, with a corresponding SW of 123€. The VCG mechanism followed closely with 596 kWh of energy traded and generating SW of 89€. However, the VCG mechanism’s SW is comparatively lower than the auction mechanism. The McAfee mechanism yielded more SW than the VCG mechanism, with a revenue of 79€for prosumers and savings of 33€for consumers. Overall, the results suggest that the auction mechanism is the most suitable mechanism for consumer-centric MGs. MG 4 is distinct from MG 3 in that it lacks EV charging points and has an equal distribution of consumers and prosumers, thus completing the energy demand of all peers. The comparative analysis for MG 4 indicates that the VCG mechanism provides the highest energy traded, with the average mechanism resulting in higher SW. The VCG mechanism benefits energy trading, regardless of the scenario, with energy trading in scenario 1 amounting to 520 kWh and scenario 3 amounting to 518 kWh, both of which are higher than any other mechanism. However, the SW generated by the VCG mechanism is lower, providing only 77€and 81€for scenario 1 and 2, respectively. In contrast, the average mechanism outperforms the VCG mechanism in terms of SW, with a potential of up to 100€SW in a day. Therefore, the average mechanism is more suitable for gaining SW during peak hours, while the VCG mechanism is more suitable for high energy traded during non-peak hours. In MG 5, the VCG mechanism resulted in the highest energy traded of 141 kWh, while the average mechanism generated the highest SW of 23€. This finding is consistent with previous MG cases. Notably, the EV-centric MG, which is MG 5, generated the least SW among all MGs. The maximum SW that MG 5 can generate in an entire day is 23€using scenario 1.

Overall, the VCG mechanism performed well in terms of energy trading and SW in all MG scenarios, except for MG 4, where the average mechanism provided higher SW. The Trade Reduction mechanism performed poorly in terms of both energy trading and SW. Additionally, the results suggest that the auction mechanism is the most suitable mechanism for consumer-centric MGs, while the VCG mechanism is more suitable for high-energy traded during non-peak hours. Finally, the EV-centric MG (MG 5) generated the least SW among all MGs, with the maximum SW generated being 23€using scenario 1.

**Fig. 7:**
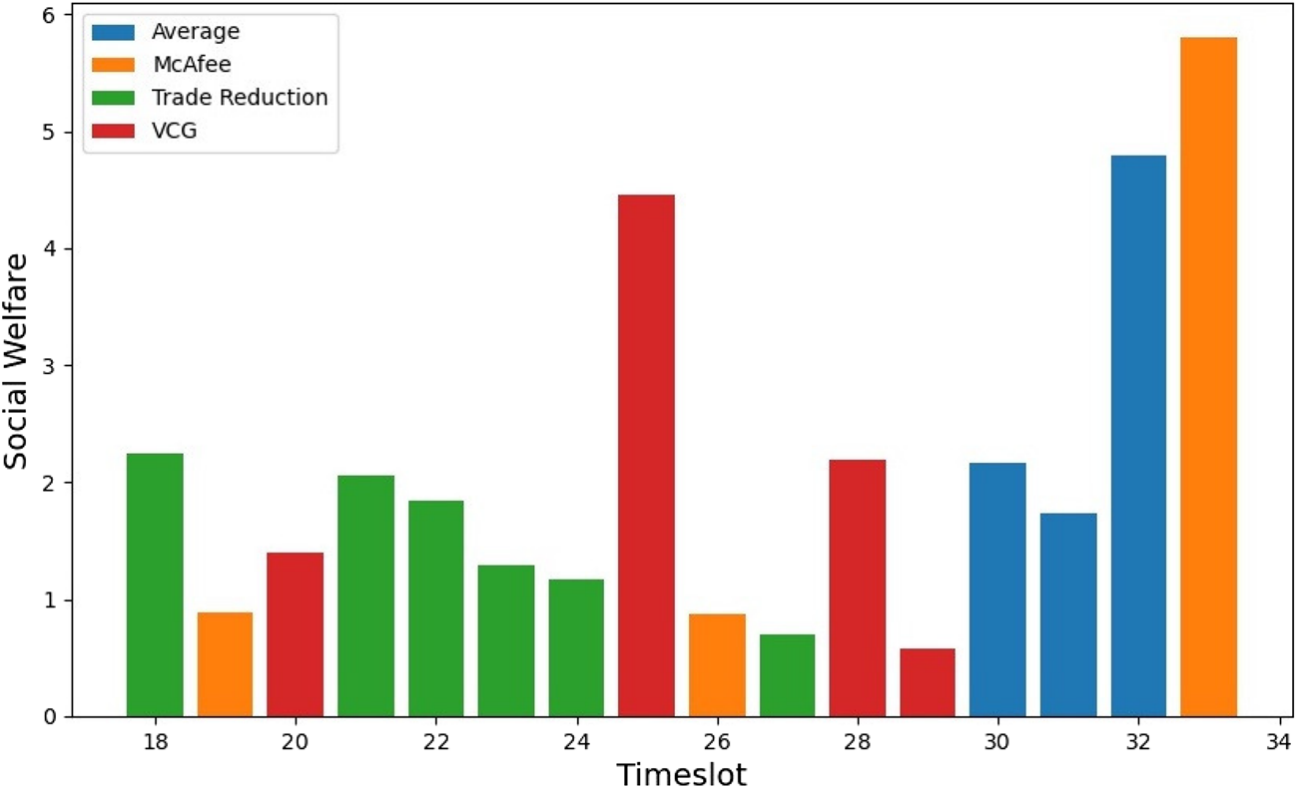
Selection of auction mechanism for each time slot based on the social welfare using the best possible scenario

**Fig. 8:**
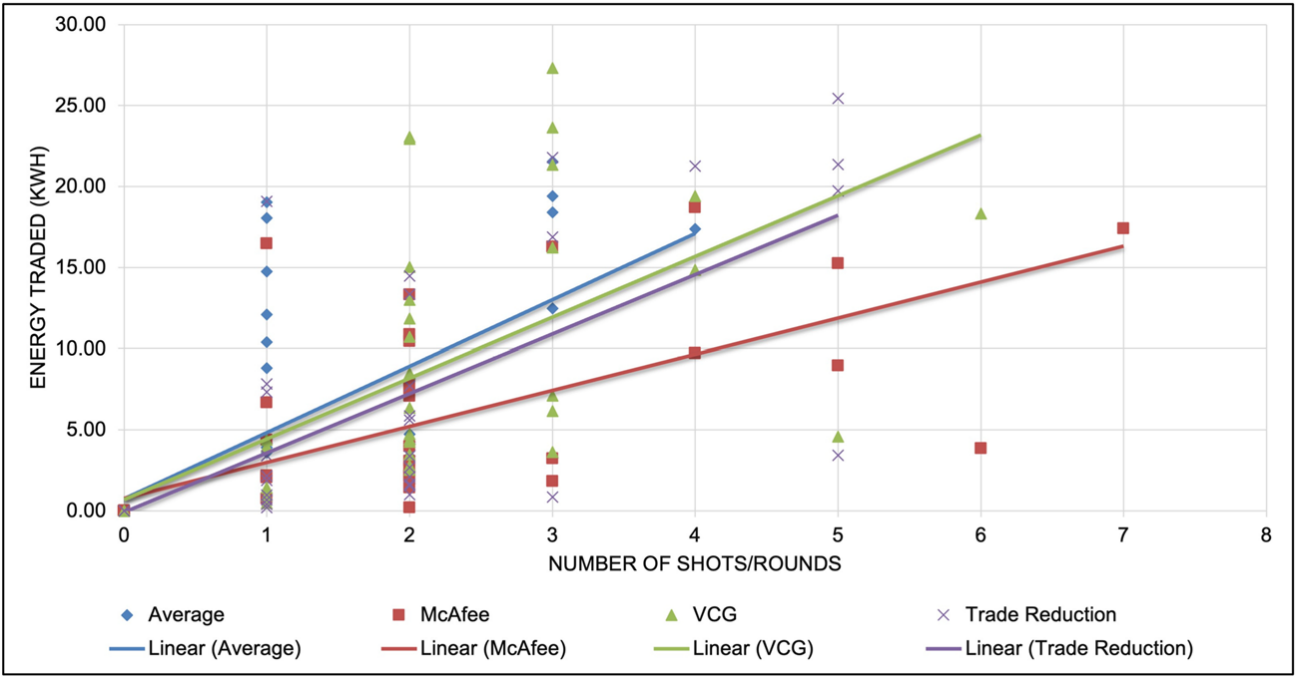
Shots v/s energy traded for various DA mechanisms

### Selection of Auction Mechanism

Figure 6 illustrates the auction mechanism selection based on the amount of energy traded for a MG with an equal distribution of consumers and prosumers. The results show that the VCG mechanism outperforms the other three mechanisms in terms of winning demand. On the other hand, Fig. 7 displays the auction mechanism selection based on SW, where Trade Reduction yields the highest SW when divided into 30-minute time intervals. It is important to note that both selections are based on scenario 3, which considers ToU and Penny Auction. A P2P market with a higher percentage of sold kWh would attract more prosumers, whereas a P2P market with a higher percentage of purchased kWh would encourage more consumer engagement. The percentage of peers cleared, i.e., the ratio of participants whose orders have been fully filled to all participants from the community aggregator’s perspective, indicates the level of satisfaction with the MG’s energy needs and surplus. A higher percentage of cleared peers implies better market performance.

At a given time interval $t_i$, the DA mechanism can execute multiple shots, as long as the total energy demand of the consumers or the surplus energy of the prosumers is non-zero. Each round is composed of various transactions, which involve orders made by a consumer to a prosumer through matching and pairing. It is important to note that this matching and pairing can also involve multiple peers. Figure 8 illustrates the energy traded by each DA mechanism through multiple shots. By simulating various shots of energy trading, we can determine the mechanism that offers the best computational efficiency. On average, the VCG mechanism delivers the highest energy traded and this observation is consistent across multiple MGs. The graph also depicts a linear model of the energy traded. However, the VCG mechanism is known to be computationally complex. Its complexity arises from the fact that it necessitates solving a nonlinear optimization problem in each auction round, which is a challenging task. Specifically, determining the optimal allocation and payment requires solving a set of simultaneous equations that may not have a closed-form solution. Therefore, numerical methods are usually employed to arrive at a solution, which is computationally intensive. Furthermore, the VCG mechanism mandates that each bidder submit a bid for every possible combination of bids from other bidders, which leads to an exponential increase in the number of bids as the number of bidders increases.

**Fig. 9:**
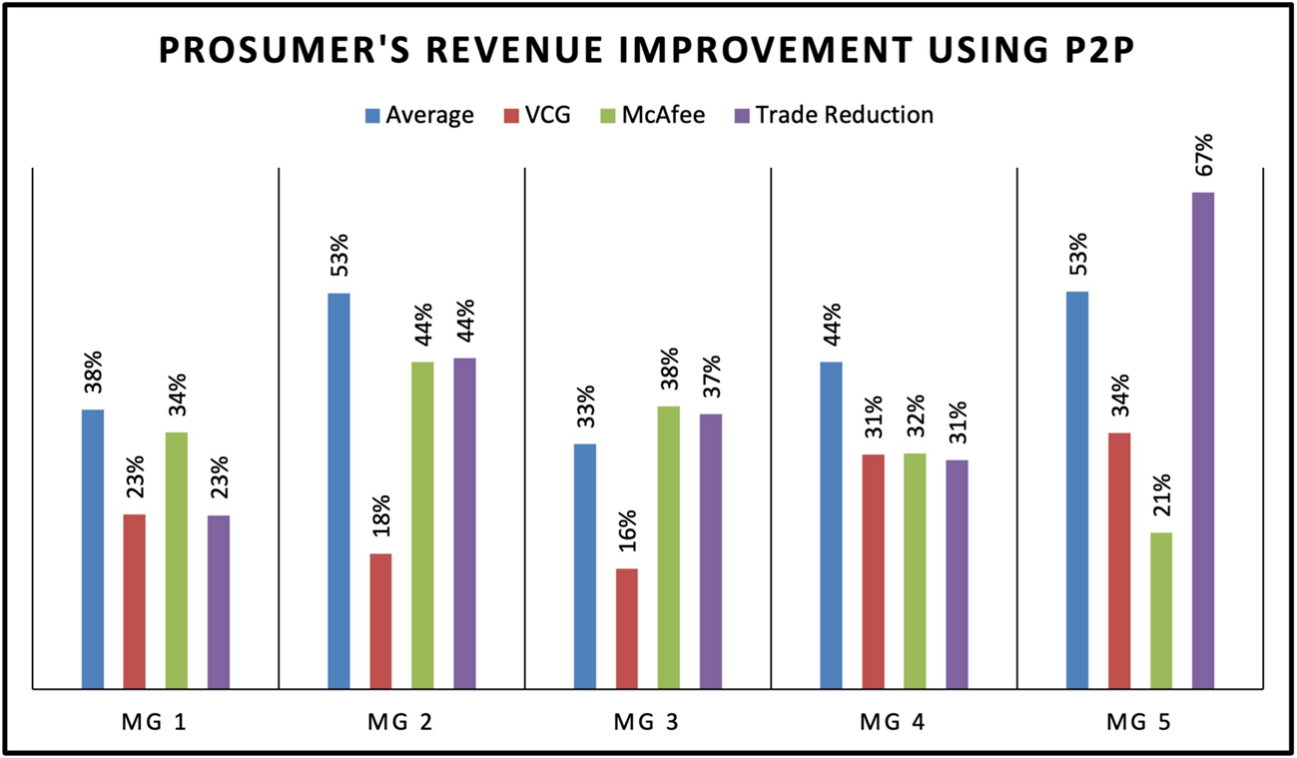
Prosumers revenue improvement using P2P

**Fig. 10:**
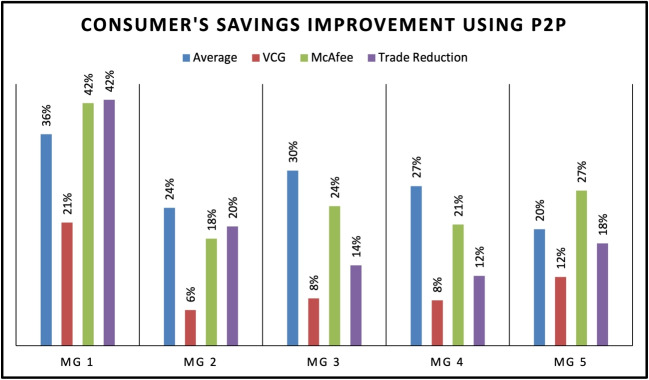
Consumer savings improvement using P2P

Figure 9 summarizes the Prosumer Revenue Improvement using P2P for five different MG using four different DA mechanisms: Average, VCG, McAfee and Trade Reduction. The MGs represent different scenarios such as Prosumer Centric, Consumer-Centric, Equal Distribution, No EV’s and EV Centric as MG 1,2,3,4 AND 5. The results show that the Trade Reduction mechanism provides the highest revenue improvement for the Consumer-Centric and Equal Distribution scenarios, while the VCG mechanism performs the best for the Prosumer Centric, No EV’s and EV Centric scenarios. The McAfee mechanism shows mixed results, with lower revenue improvement for some scenarios and higher improvement for others. Overall, the results suggest that the selection of the DSA mechanism should be based on the MG’s specific scenario and the market participants’ objective. Figure 10 shows the percentage of consumer savings improvement using P2P mechanisms in five different MGs. The results indicate that the highest consumer savings improvement is observed in the prosumer-centric approach using the McAfee mechanism, with an average improvement of 36%. The VCG mechanism also shows significant improvement with an average of 21%. For MGs with equal distribution and no EVs, the trade reduction mechanism provides better consumer savings improvement compared to other mechanisms. In contrast, for MGs with EVs, the trade reduction mechanism performs the best, providing an average of 18% consumer savings improvement. Just like prosumers improvement, the results indicate that the selection of P2P mechanisms should be based on the MG’s characteristics and requirements to optimize consumer savings improvement. Therefore, the proposed trading algorithm allows aggregator to change the auction mechanism depending on the nature of MG at each time intreval.

**Fig. 11:**
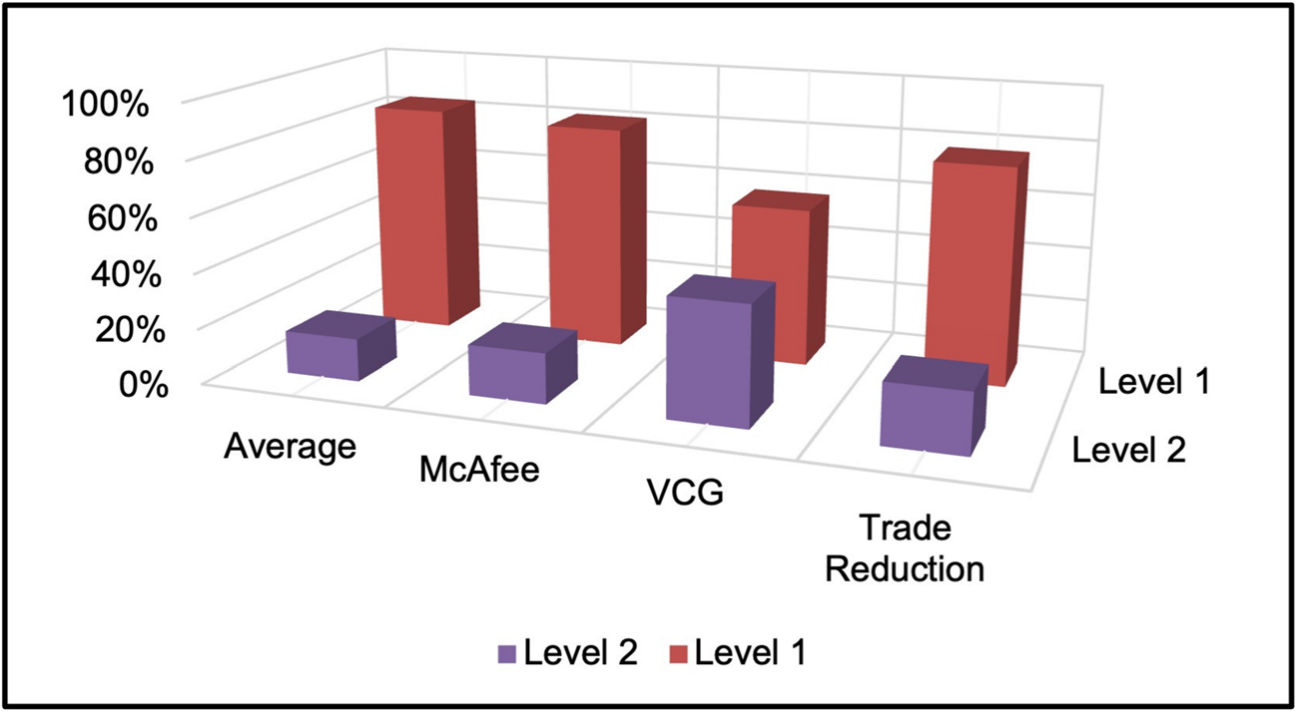
Energy traded in level 1(P2P) and level 2(MG to MG)

Furthermore, once energy trading is completed at this level, the trading algorithm shifts to level 2. In level 2, these five MGs trade with each other to fulfil the energy demand of the consumers and sell the surplus energy of the prosumers. Each MG acts as a peer and goes through the trading algorithm. However, it was observed that P2P energy trading from MG to MG was inefficient due to the lesser number of peers. An efficient method of trading between MGs is instead of considering one MG as one peer; we can count all the consumers acting as buyers and the prosumers acting as sellers as peers. This way, the number of players increases and the auction mechanism will give better results. With the simulations, results indicate that the majority of energy is traded at this level 1, with an average of 85% Fig. 11. The Trade Reduction mechanism performs best in level 1, with 78% of energy traded through P2P, followed by the Average and McAfee mechanisms. The VCG mechanism performs the worst in level 1, with only 57% of energy traded through P2P. Level 2 represents MG-to-MG energy trading and the results show that this level contributes a relatively smaller portion of energy trading, with an average of 15%. The VCG mechanism performs the best in level 2, with 43% of energy traded through MG-to-MG trading, followed by McAfee and Trade Reduction mechanisms.

## Conclusions and Future Work

This study explored a DA trading algorithm designed for P2P energy trading within a diverse range of MGs. We delved into the four major DA mechanisms in P2P energy trading: Average, McAfee, Trade Reduction, and VCG Mechanism. Our aim was to allow users the flexibility to express their willingness to choose the most suitable DA and their time-specific preferences, setting it apart from other studies. Our algorithm considered different MGs - prosumer-centric, consumer-centric, EV-centric, equally distributed, and no EVs. Proposed P2P energy trading algorithm that incorporates all four auction mechanisms, along with bidding strategies, preference parameters and ToU tariffs to improve the efficiency and effectiveness of energy trading was tested. The results reveal that average and VCG mechanisms are best suitable for most types of MGs. Among them, the VCG mechanism demonstrated robust results in terms of energy trading and SW across most MG scenarios. A notable exception was observed in MG 4, where the average mechanism outperformed VCG in terms of SW. On a more granular level, the VCG mechanism showed excelling during non-peak hours, while the average mechanism was found to be optimal during peak demand periods. Interestingly, the results indicated that EV-centric MGs (MG 5) achieved the lowest social welfare across all other MGs. In conclusion, our proposed novel P2P energy trading algorithm provides a comprehensive solution that takes into account various user preferences and identifies the most suitable auction mechanisms for different types of MGs at each time interval.

Future work will investigate network constraints such as voltage management and power factor. Additionally, we aim to delve into the potential of blockchain technology for P2P energy trading. Blockchain, being inherently secure due to its decentralized and immutable nature, is viewed as a promising solution to address the security challenges in P2P energy trading. It could offer a transparent platform for energy transactions, potentially reducing transaction costs and enhancing the efficiency of the trading process. Unauthorized access, data tampering, and false data injection are concerns that blockchain can mitigate, ensuring the integrity and reliability of energy trades. Establishing robust encryption, authentication protocols, and a comprehensive cybersecurity framework will remain crucial as we move forward.

## Data Availability

The data sets generated during and/or analysed during the current study are available in the [IEEE PES test feeder] repository (https://cmte.ieee.org/pes-testfeeders/resources/).

## Nomenclature

$\alpha _{c,p}$: Preference parameter of consumer for a prosumer
$\alpha _{p,c}$: Preference parameter of prosumer for a consumer
$\chi _{n}$: True bid by the player
$\epsilon$: Fixed increment price of the bid
$\kappa _{n}$: False bid by the player
*AP*: Asking price of a time slot
$b_{c_i}$: Bid price offered by consumer for buying energy
*BP*: Buying price of a time slot
*C*: Set of all consumers
$C_{E}$: Cost per unit of energy
$c_{p_j}$: Valuation function of seller *j*
$D_{c_i}$: Energy demand of consumer $C_i$
$D_{v}$: Energy demand of Electric vehicle *v*
*k*: Breakeven Index
$K^{off-peak}$: ToU tariff rate during off-peak hours
$K^{peak}$: ToU tariff rate during peak hours
$K^{shoulder}$: ToU tariff rate during shoulder hours
$K^{t}$: ToU tariff rate at time interval *t*
*N*: Set of all peers
*P*: Set of all prosumers
$$P_{Eq}$$: Equilibrium price
$P_{fit}$: Feed-in tariff rate, the price at which the prosumer sells energy back to the grid
$P_{grid}$: Price at which the consumer buys energy from the grid
$$P_{\max }$$: Ceiling price
$$P_{\min }$$: Floor price
$p_{p_j}$: Ask price set by prosumer for selling their excess energy
$Q_{load}(t)$: Total demand of the peers
$Qty_{Grid}$: Quantity of energy exchanged with the grid
$Qty_{p2p}$: Quantity of P2P energy traded
*R*: Total revenue
$S_{p_j}$: surplus energy that prosumer $P_j$
*SW*: Social Welfare
$t_i$: Each discrete time slot in which the trading occurs
*TP*: Trading price of a time slot
$U_{cons}$: Utility function of the consumer
$U_{n}$: Utility of player n
$U_{pros}$: Utility function of the prosumer
*v*: Electric Vehicle
$v_{c_i}$: Valuation function of buyer *i*
$x_{c_i, p_j}$: A binary decision variable. It equals 1 if the consumer and prosumer trade successfully and 0 otherwise.

## Glossary

*BB*: Balanced Budget.
*CE*: Computational Efficiency.
*DA*: Double Auction.
*DER*: Distributed Energy Resources.
*DSO*: Distribution System Operators.
*EE*: Economic Efficiency.
*EV*: Electric Vehicle.
*IC*: Incentive Compatibility.
*IR*: Individual rationality.
*MG*: Microgrid.
*P*2*P*: Peer-to-Peer.
*RE*: Renewable Energy.
*SW*: Social Welfare.
*ToU*: Time of Usage.
*TR*: Trade Reduction.
*VCG*: Vickrey-Clarke-Groves.

## Code Availability

The code can be shared upon request.
